# Voltammetric determination of hydrochlorothiazide at a modified carbon paste electrode with polypyrrole nanotubes

**DOI:** 10.5599/admet.1706

**Published:** 2023-03-15

**Authors:** Arefeh Mohammadnavaz, Fariba Garkani-Nejad

**Affiliations:** 1 Department of Chemistry, Graduate University of Advanced Technology, Kerman, Iran; 2 Environment Department, Institute of Science and High Technology and Environmental Sciences, Graduate University of Advanced Technology, Kerman, Iran

**Keywords:** Carbon paste electrodes, polypyrrole nanotubes, modified electrode, voltammetry, hydrochlorothiazide

## Abstract

In this paper, the electrochemical behavior of hydrochlorothiazide (HCTZ) is described using carbon paste electrodes modified with polypyrrole nanotubes (PPy-NTs/CPEs) at pH value 7. Experiments revealed that the presence of HCTZ greatly impacts the electrochemical behavior of modified CPEs. The synthesized PPy-NTs were utilized as a sensing material for the electrochemical detection of HCTZ and were investigated by cyclic voltammetry (CV), differential pulse voltammetry (DPV) and chronoamperometry. The key experiment conditions, including supporting electrolyte and electrolyte pH, were studied and optimized. Under optimized conditions, the prepared sensor displayed the linear relationships for the concentrations of HCTZ from 5.0 to 400.0 μM (R^2^ = 0.9984). The detection limit of the PPy-NTs/CPEs sensor was found to be 1.5 μM using the DPV method. The PPy-NTs is highly selective, stable and sensitive for the determination of HCT. Therefore, we believe the newly prepared PPy-NTs material can be useful for different electrochemical applications.

## Introduction

Hypertension (high blood pressure) adds to the workload of the heart and arteries. If it continues long, the heart and arteries may not function properly. This can damage the blood vessels of the brain, heart, and kidneys, resulting in a stroke, heart attack or kidney failure. These problems may be less likely to occur if blood pressure is controlled. Hydrochlorothiazide (HCTZ) combination is used to treat hypertension. Hydrochlorothiazide is a thiazide-type diuretic that has been used clinically for more than half a century. The drug has been widely used to treat hypertension globally and is relatively safe. Hydrochlorothiazide acts on the distal convoluted tubules and inhibits the sodium chloride co-transporter system. This action leads to a diuretic action that lowers blood pressure, but there is also a potassium loss in the urine. Hydrochlorothiazide is also helpful in removing the excess water from the body; however, calcium retains in the body. Moreover, as a therapeutic option for congestive heart failure, diabetes insipidus, renal tubular acidosis, and symptomatic edema. In addition, it prevents kidney stones [1-5]. All these aspects encourage investigating the electrochemical features of HCTZ on novel materials, and it has been studied with an increasing trend in recent research activities around the world.

The various techniques available for the selective and sensitive detection of HCTZ include HPLC, capillary zone electrophoretic, spectrophotometric/HPLC and electrochemical methods [6-11]. Electrochemistry-based methods can be employed considering their lower cost, speediness, portability, reasonable selectivity, simple preparation process, suitable accuracy, and precision for the analysis of biological compounds, gas pollutants, drugs, food and water pollutants compounds [12-22]. The chemical modification of inert substrate electrodes offers significant advantages in the design and development of electrochemical sensors. The properties of chemically modified electrodes that have driven their development include increased selectivity and sensitivity, chemical and electrochemical stability, larger usable potential windows, and resistance to fouling [23-35]. The incorporation of nanomaterials has had a great impact on the development of electrochemical sensors [36-40]. Significant progress has been made toward synthesizing nanomaterials with controllable morphologies, dimensions, surface charges, and physicochemical properties [41-62].

Conducting polymers (CPs) derivatives of polypyrrole nanotubes (PPy-NTs) are 1-D nanostructured materials and are considered as one of the ideal candidates due to their large usage in various industrial applications and tremendous physico-chemical properties, such as high electrical conductivity, good mechanical stability, nanoscale particle size, lightweight, and wide surface area. PPy-NTs has an excellent material for device fabrication in electrochemical sensors or biochemical fields due to their small diameter with an average length, which may allow more scope for proper chemical interaction with a doping material and enhance the surface area and sensitivity compared to the bulk material. PPy-NTs show a high conductivity due to extended π conjugation and a tunable doping process that promotes the electrochemical oxidation activity [63,64].

Based on the above-mentioned information and in light of the information presented, the current study was based on the development and application of PPy-NTs/CPEs for the voltammetric determination of HCTZ. Inspired by the aforementioned discussions, we modified the bare electrodes with PPy-NTs, which are applied to improve the selectivity and sensitivity of the electrochemical sensor. Hence, the PPy-NTs were synthesized and developed as a highly sensitive and selective platform for detecting HCTZ. The constructed sensor has good performance characteristics, simplicity of preparation, high selectivity, stability, wide linear range and a small limit of detection. It was successfully applied for the voltammetry determination of HCTZ in biological samples.

## Experimental

### Equipment and materials

In order to do electrochemical tests at ambient temperature, we utilized the Auto-lab potentiostat /galvanostat (PGSTAT 302N, Eco Chemie, the Netherlands) with GPES (General Purpose Electrochemical System-version 4.9) software to control the system. Electrochemical measurements were performed at room temperature in a conventional electrochemical cell with a PPy-NTs/CPE as the working electrode, 3.0 M Ag/ AgCl/KCl as a reference electrode (Azar Electrode, Urmia, Iran) and platinum wire as a counter electrode (Azar Electrode, Urmia, Iran). Moreover, pH was measured using the Metrohm 713 pH-meter with a glass electrode (Switzerland). Hydrochlorothiazide and all other solutions used during the procedure were prepared by reagent-grade chemicals from Merck and Sigma-Aldrich and deionized water was supply from Millipore, Germany. Orthophosphoric acid was utilized to prepare the phosphate buffer solutions (PBSs), and sodium hydroxide was used to adjust the desired pH values (pH range between 2.0 and 9.0).

### Preparation of PPy nanotubes

**Figure 1.**
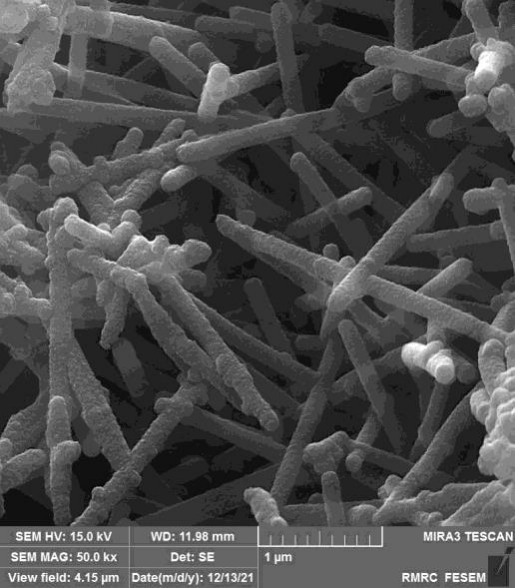
FE-SEM image of PPy nanotubes.

Polypyrrole nanotubes (Ppy-NTs) were prepared by the oxidation of pyrrole monomer with iron(III) chloride in the presence of a structure-guiding agent, methyl orange. In a typical synthesis, 0.784 g (2.3 mmol) methyl orange and 3.888 g (23 mmol) FeCl_3_ were dissolved into 480 mL of deionized water. Then 0.84 mL (12.1 mmol) of pyrrole was added to the solution and stirred for 24 h at room temperature. The formed PPy precipitate was washed with deionized water/ethanol several times until the filtrate was colorless and neutral and finally dried under a vacuum atmosphere at 65 °C for 20 h. Figure 1 shows the FE-SEM image of PPy nanotubes.

### Preparation and surface modification of electrode

To prepare PPy-NTs/CPE, 0.95 g graphite powder and 0.05 g PPy-NTs were mixed. Next, a suitable amount of paraffin oil was poured into the resulting mixture, followed by mixing well for 30 min to obtain a uniformly wetted paste. An appropriate amount of the paste was tightly packed into a glass tube and a copper wire was positioned over the carbon paste to make electrical contact.

## Results and discussion

### Electrochemical behavior of HCTZ on polypyrrole nanotubes

**Scheme 1.**
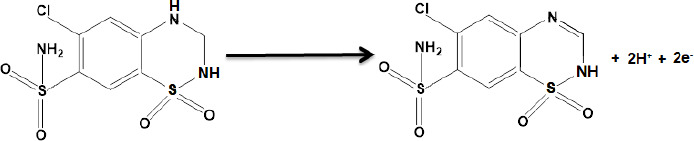
Mechanism for the oxidation of HCTZ at the surface of PPy-NTs/CPE.

According to our knowledge, the electrooxidation of HCTZ is closely related to the pH value of the solution. So, the effect of pH was investigated using the differential pulse voltammetry (DPV) method. The results show that the oxidation peak current increased slowly from pH 2.0 to 7.0, and then the current conversely decreased when the pH value increased from 7.0 to 9.0. According to obtained results, pH 7.0 was chosen as the optimal experimental condition for other experiments. The electrochemical reaction of HCTZ involves two electrons and two protons, according to Scheme 1.

**Figure 2.**
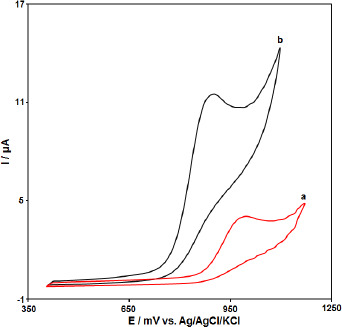
Cyclic voltammograms of a) CPE and b) PPy-NTs/CPE in the presence of 200.0 μM HCTZ at a pH 7.0 of 0.1 M PBS, respectively.

The electrochemical behavior of the CPE, PPy-NTs/CPE was studied by the cyclic voltammetry (CV) technique in the 0.1 M phosphate buffer (pH=7.0) as the supporting electrolyte at a scan rate of 50 mV s^−1^ (Figure 2). As shown in Figure 2, in comparison to the bare CPE (a), PPy-NTs/CPE (b) presents a well-defined Irreversible oxide peak with a higher current signal (HCTZ concentration equal to 200.0 μM).

### Role of variable scan rates

**Figure 3.**
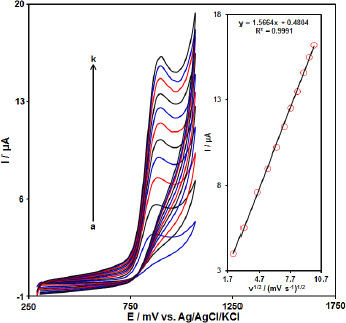
Linear sweep voltammograms of HCTZ (200 μM) at PPy-NTs/CPE at different scan rates of a) 5, b) 10, c) 20, d)30, e) 40, f) 50, g) 60, h) 70, i) 80, j) 90 and k) 100 mV/s in 0.1 M PBS (pH 7.0). Insert: Plot of *I*p versus *v* ^1/2^ for the oxidation of HCTZ at PPy-NTs/CPE.

The effect of the potential scan rates (5-100 mV s^-1^) on the electrochemical oxidation of HCTZ was studied by linear sweep voltammograms (LSV). Figure 3 shows the LSV of 200.0 μM of HCTZ in the 0.1 M phosphate buffer solution at the PPy-NTs/CPE. These results show that the anodic current increases with increasing scan rate. The oxidation current of HCTZ increased linearly with the square root of the scan rate (Figure 3, Inset), demonstrating a diffusion-controlled electrochemical process.

### Chronoamperometric analysis

**Figure 4.**
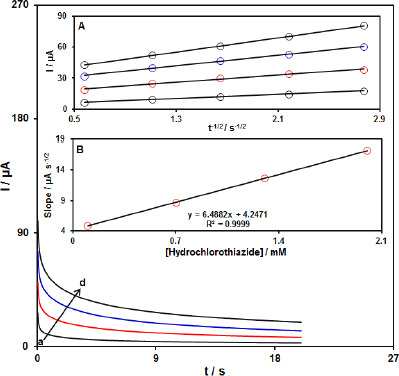
Chronoamperograms obtained at the PPy-NTs/CPE in the presence of a) 0.1, b) 0.7, c) 1.3 and d) 2.0 mM HCTZ in the 0.1 M buffer solution (pH 7.0). A) Plot of *I* versus *t*^-1/2^ for electrooxidation of HCTZ obtained from chronoamperoms a–d. B**)** Plot of slope from straight lines versus HCTZ level.

The chronoamperometric measurements of HTCZ at the PPy-NTs/CPE surface were done to estimate the apparent diffusion coefficient of HTCZ. Figure 4 shows the current-time profiles obtained by setting the working electrode potential at 950 mV for different concentrations of HTCZ. At long enough experimental times (*t*=0.3-3s), where the electron transfer reaction rate of HTCZ is more than its diffusion rate toward the working electrode surface, the current is diffusion controlled. Figure 4, inset A, shows the experimental plots of *I* versus *t*^-1/2^ with the best fit for different concentrations of HTCZ employed. The slopes of the resulting straight lines were then plotted versus the HTCZ concentration (Figure 4, inset B). Based on the Cottrell equation [65], the slope of this plot (Figure 4 inset B) can be used to estimate the apparent diffusion coefficient of HTCZ. From the slope of this plot (11.644 A s^1/2^ mM^-1^), the value of diffusion coefficient was found to be 1.7w10^-6^ cm s^1^.

### DPV analysis of HCTZ

**Figure 5.**
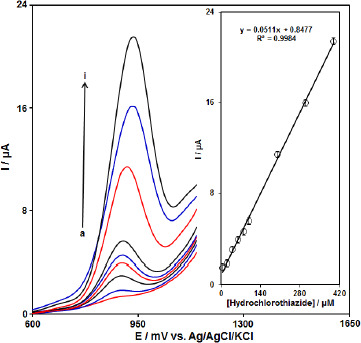
DPV curves of PPy-NTs/CPE in the 0.1 M buffer solution (pH 7.0) containing different concentrations of HCTZ. a-i corresponds to 5.0, 20.0, 40.0, 60.0, 80.0, 100.0, 200.0, 300.0 and 400.0 μM HCTZ. Inset: Plots of oxidation peak.

DPV was used for the determination of HCTZ at PPy-NTs/CPE due to its high sensitivity. The DPV responses for different concentrations of HCTZ are illustrated in Figure 5. The linear range was found to be 5.0 μM to 400.0 μM. The linear equation was *I*p (μA)=0.8477-0.0511 *C*_HCTZ_ (μM) with a correlation coefficient of 0.9984. The detection limit was 1.5 μM (S/N=3).

## Conclusion

A novel electrochemical protocol using PPy-NTs/CPE was fabricated for the sensitive determination of HCTZ. The modified electrode electrocatalytically oxidizes the HCTZ at a less positive potential with an increased oxidation current. The electrocatalytic oxidation current of HCTZ was linearly increased with the increased concentration of HCTZ. The sensor under the optimized circumstances possessed a fast current response to HCTZ, with a linear dynamic range between 5.0-400.0 μM, a thin limit of detection of 1.5 μM, and an appreciable sensitivity of 0.0511 μA/μM. According to the analyses, the modified electrode demonstrated acceptable electrocatalytic activities and sensitivity. Also, excellent features, like a wide linear range, low detection limit, high reproducibility and repeatability and longtime stability, proved the successful application of this sensor for the determinations of HCTZ.

## References

[1] Nezhadali A. Mojarrab M.. Computational study and multivariate optimization of hydrochlorothiazide analysis using molecularly imprinted polymer electrochemical sensor based on carbon nanotube/polypyrrole film. Sensors and Actuators B: Chemical 190 (2014) 829-837. https://doi.org/10.1016/j.snb.2013.08.086 10.1016/j.snb.2013.08.086

[2] Lal R. Tahira A. Khand A.A. Qureshi I.N. Mangi J. Lakho S.A. Aftab U. Lal B. Basha S. Karami A.M. Al-Saeedi S.L.. Flower-like CuO/polyaniline composite for electrochemical determination of hydrochlorothiazide. Bulletin of Materials Science 44 (2021) 1-8. https://doi.org/10.1007/s12034-021-02528-w 10.1007/s12034-021-02528-w

[3] Ernst M.E. Fravel M.A.. Thiazide and the thiazide-like diuretics: review of hydrochlorothiazide, chlorthalidone, and indapamide. American Journal of Hypertension 35(7) (2022) 573-586. https://doi.org/10.1093/ajh/hpac048 10.1093/ajh/hpac048 35404993

[4] Shahabi Z. Zare-Shahabadi V. Sayyahi S. Burromand-Piroz J.. Novel CuO/polymethylenedisulfide nanocomposite for high performance electrocatalytic determination of hydrochlorothiazide in real samples. Journal of Porous Materials 29(4) (2022) 1123-1135. https://doi.org/10.1007/s10934-022-01236-y 10.1007/s10934-022-01236-y

[5] Núñez-Acevedo B. Domínguez-Ortega J. Rodríguez-Jiménez B. Kindelan-Recarte C. Pérez-Fernández M.A.. Severe and rare adverse reaction to hydrochlorothiazide. Revista Alergia México 65(4) (2018) 442-445. https://doi.org/10.29262/ram.v65i4.363 10.29262/ram.v65i4.363 30602216

[6] Hemdan A. Al-Tannak N.F. Mohamed E.H.. Development of a multivariate model with desirability-based optimization for determination of atenolol and hydrochlorothiazide by eco-friendly HPLC method with fluorescence detection. Journal of Separation Science 45(4) (2022) 824-831. https://doi.org/10.1002/jssc.202100711 10.1002/jssc.202100711 34910855

[7] Haque S.M.. Box–Behnken experimental design for optimizing the HPLC method to determine hydrochlorothiazide in pharmaceutical formulations and biological fluid. Journal of Molecular Liquids 352 (2022) 118708. https://doi.org/10.1016/j.molliq.2022.118708 10.1016/j.molliq.2022.118708

[8] Ahmed H.M. Belal T.S. Shaalan R.A. El Yazbi F.A. Elonsy S.M.. Validated capillary zone electrophoretic method for simultaneous analysis of benazepril in combination with amlodipine besylate and hydrochlorothiazide. Acta Chromatographica 32(4) 2020 219-227. https://doi.org/10.1556/1326.2019.00686 10.1556/1326.2019.00686

[9] Tiris G. Mehmandoust M. Lotfy H.M. Erk N. Joo S.W. Dragoi E.N. Vasseghian Y.. Simultaneous determination of hydrochlorothiazide, amlodipine, and telmisartan with spectrophotometric and HPLC green chemistry applications. Chemosphere 303 (2022) 135074. https://doi.org/10.1016/j.chemosphere.2022.135074 10.1016/j.chemosphere.2022.135074 35667505

[10] Silva E.F. Tanaka A.A. Fernandes R.N. Munoz R.A.A. da Silva I.S.. Batch injection analysis with electrochemical detection for the simultaneous determination of the diuretics furosemide and hydrochlorothiazide in synthetic urine and pharmaceutical samples. Microchemical Journal 157 (2020) 105027. https://doi.org/10.1016/j.microc.2020.105027 10.1016/j.microc.2020.105027

[11] Khanfar M.F. Abu-Nameh E.S. Saket M.M. Al Khateeb L.T. Al Ahmad A. Asaad Z. Salem Z. Alnuman N.. Detection of Hydrochlorothiazide, Sulfamethoxazole, and Trimethoprim at Metal Oxide Modified Glassy Carbon Electrodes. International Journal of Electrochemical Science 15 (2020) 1771-1787. https://doi.org/10.3390/ma13112521 10.3390/ma13112521

[12] Karimi-Maleh H. Karimi F. Orooji Y. Mansouri G. Razmjou A. Aygun A. Sen F.. A new nickel-based co-crystal complex electrocatalyst amplified by NiO dope Pt nanostructure hybrid; a highly sensitive approach for determination of cysteamine in the presence of serotonin. Scientific Reports 10(1) (2020) 1-13. https://doi.org/10.1038/s41598-020-68663-2 10.1038/s41598-020-68663-2 32678156 PMC7366926

[13] Lohrasbi-Nejad A.. Electrochemical strategies for detection of diazinon. Journal of Electrochemical Science and Engineering 12(6) (2022) 1041-1059. https://doi.org/10.5599/jese.1379 10.5599/jese.1379

[14] Mohanraj J. Durgalakshmi D. Rakkesh R.A. Balakumar S. Rajendran S. Karimi-Maleh H.. Facile synthesis of paper based graphene electrodes for point of care devices: A double stranded DNA (dsDNA) biosensor. Journal of Colloid and Interface Science 566 (2020) 463-472. https://doi.org/10.1016/j.jcis.2020.01.089 10.1016/j.jcis.2020.01.089 32032811

[15] Mazloum-Ardakani M. Beitollahi H. Taleat Z. Naeimi H. Taghavinia N.. Selective voltammetric determination of d-penicillamine in the presence of tryptophan at a modified carbon paste electrode incorporating TiO_2_ nanoparticles and quinizarine. Journal of Electroanalytical Chemistry 644(1) (2010) 1-6. https://doi.org/10.1016/j.jelechem.2010.02.034 10.1016/j.jelechem.2010.02.034

[16] Mustafa Y.F. Chehardoli G. Habibzadeh S. Arzehgar Z.. Electrochemical detection of sulfite in food samples. Journal of Electrochemical Science and Engineering 12(6) (2022) 1061-1079. https://doi.org/10.5599/jese.1555 10.5599/jese.1555

[17] Miraki M. Karimi-Maleh H. Taher M. A. Cheraghi S. Karimi F. Agarwal S. Gupta V.K.. Voltammetric amplified platform based on ionic liquid/NiO nanocomposite for determination of benserazide and levodopa. Journal of Molecular Liquids 278 (2019) 672-676. https://doi.org/10.1016/j.molliq.2019.01.081 10.1016/j.molliq.2019.01.081

[18] Velicky M. Rodgers A.N. Dryfe R.A. Tam K.. Use of voltammetry for in vitro equilibrium and transport studies of ionisable drugs. ADMET and DMPK 2(3) (2014) 143-156. https://doi.org/10.5599/admet.2.3.22 10.5599/admet.2.3.22

[19] Kazemipour M. Ansari M. Mohammadi A. Beitollahi H. Ahmadi R.. Use of adsorptive square-wave anodic stripping voltammetry at carbon paste electrode for the determination of amlodipine besylate in pharmaceutical preparations. Journal of Analytical Chemistry 64 (2009) 65-70. https://doi.org/10.1134/S1061934809010134 10.1134/S1061934809010134

[20] Mohabis R.M. Fazeli F. Amini I. Azizkhani V.. An overview of recent advances in the detection of ascorbic acid by electrochemical techniques. Journal of Electrochemical Science and Engineering 12(6) (2022) 1081-1098. https://doi.org/10.5599/jese.1561 10.5599/jese.1561

[21] Karimi-Maleh H. Sheikhshoaie M. Sheikhshoaie I. Ranjbar M. Alizadeh J. Maxakato N.W. Abbaspourrad A.. A novel electrochemical epinine sensor using amplified CuO nanoparticles and an-hexyl-3-methylimidazolium hexafluorophosphate electrode. New Journal of Chemistry 43(5) (2019) 2362-2367. https://doi.org/10.1039/C8NJ05581E 10.1039/C8NJ05581E

[22] Meoipun A. Kaewjua K. Chailapakul O. Siangproh W.. A simple and fast flow injection amperometry for the determination of methimazole in pharmaceutical preparations using an unmodified boron-doped diamond electrode. ADMET and DMPK (2023). https://doi.org/10.5599/admet.1584 10.5599/admet.1584 PMC10262230 37325121

[23] Taleat Z. Ardakani M. M. Naeimi H. Beitollahi H. Nejati M. Zare H. R.. Electrochemical behavior of ascorbic acid at a 2, 2'-[3, 6-dioxa-1, 8-octanediylbis (nitriloethylidyne)]-bis-hydroquinone carbon paste electrode. Analytical Sciences 24(8) (2008) 1039-1044. https://doi.org/10.2116/analsci.24.1039 10.2116/analsci.24.1039 18689946

[24] Jahani P.M.. Flower-like MoS_2_ screen-printed electrode based sensor for the sensitive detection of sunset yellow FCF in food samples. Journal of Electrochemical Science and Engineering 12(6) (2022) 1099-1109. https://doi.org/10.5599/jese.1413 10.5599/jese.1413

[25] Alavi-Tabari S.A. Khalilzadeh M.A. Karimi-Maleh H.. Simultaneous determination of doxorubicin and dasatinib as two breast anticancer drugs uses an amplified sensor with ionic liquid and ZnO nanoparticle. Journal of electroanalytical chemistry 811 (2018) 84-88. https://doi.org/10.1016/j.jelechem.2018.01.034 10.1016/j.jelechem.2018.01.034

[26] Brett C.M.. Electrochemical impedance spectroscopy in the characterisation and application of modified electrodes for electrochemical sensors and biosensors. Molecules 27(5) (2022) 1497. https://doi.org/10.3390/molecules27051497 10.3390/molecules27051497 35268599 PMC8911593

[27] Mohammadi S.Z. Mousazadeh F. Mohammadhasani-Pour M.. Electrochemical detection of folic acid using a modified screen printed electrode. Journal of Electrochemical Science and Engineering 12(6) (2022) 1111-1120. https://doi.org/10.5599/jese.1360 10.5599/jese.1360

[28] Raoof J.B. Ojani R. Beitollahi H.. Electrocatalytic determination of ascorbic acid at chemically modified carbon paste electrode with 2, 7-bis (ferrocenyl ethynyl) fluoren-9-one. International Journal of Electrochemical Science 2(7) (2007) 534-548.

[29] Sanko V. Şenocak A. Tümay S. O. Orooji Y. Demirbas E. Khataee A.. An electrochemical sensor for detection of trace-level endocrine disruptor bisphenol A using Mo_2_Ti_2_AlC_3_ MAX phase/MWCNT composite modified electrode. Environmental Research 212 (2022) 113071. https://doi.org/10.1016/j.envres.2022.113071 10.1016/j.envres.2022.113071 35346651

[30] Karimi-Maleh H. Shojaei A.F. Tabatabaeian K. Karimi F. Shakeri S. Moradi R.. Simultaneous determination of 6-mercaptopruine, 6-thioguanine and dasatinib as three important anticancer drugs using nanostructure voltammetric sensor employing Pt/MWCNTs and 1-butyl-3-methylimidazolium hexafluoro phosphate. Biosensors and Bioelectronics 86 (2016) 879-884. https://doi.org/10.1016/j.bios.2016.07.086 10.1016/j.bios.2016.07.086 27494812

[31] Saghiri S. Ebrahimi M. Bozorgmehr M.. Electrochemical Amplified Sensor with Mgo Nanoparticle and Ionic Liquid: A Powerful Strategy for Methyldopa Analysis. Chemical Methodologies 5(3) (1999) 234-239. https://doi.org/10.22034/chemm.2021.128530 10.22034/chemm.2021.128530

[32] Eren T. Atar N. Yola M.L. Karimi-Maleh H.. A sensitive molecularly imprinted polymer based quartz crystal microbalance nanosensor for selective determination of lovastatin in red yeast rice. Food chemistry 185 (2015) 430-436. https://doi.org/10.1016/j.foodchem.2015.03.153 10.1016/j.foodchem.2015.03.153 25952889

[33] Saghiri S. Ebrahimi M. Bozorgmehr R.. NiO nanoparticle/1-hexyl-3-methylimidazolium hexafluorophosphate composite for amplification of epinephrine electrochemical sensor. Asian Journal of Nanosciences and Materials 4(1) (2021) 46-52. https://doi.org/10.26655/AJNANOMAT.2021.1.4 10.26655/AJNANOMAT.2021.1.4

[34] Beitollahi H. Mohammadi S.Z. Safaei M. Tajik S.. Applications of electrochemical sensors and biosensors based on modified screen-printed electrodes: a review. Analytical Methods 12(12) (2020) 1547-1560. https://doi.org/10.1039/C9AY02598G 10.1039/C9AY02598G

[35] Karimi-Maleh H. Darabi R. Shabani-Nooshabadi M. Baghayeri M. Karimi F. Rouhi J. Alizadeh M. Karaman O. Vasseghian Y. Karaman C.. Determination of D&C Red 33 and Patent Blue V Azo dyes using an impressive electrochemical sensor based on carbon paste electrode modified with ZIF-8/g-C3N4/Co and ionic liquid in mouthwash and toothpaste as real samples. Food and Chemical Toxicology 162 (2022) 112907. https://doi.org/10.1016/j.fct.2022.112907 10.1016/j.fct.2022.112907 35271984

[36] Azimi S. Amiri M. Imanzadeh H. Bezaatpour A.. Fe_3_O_4_@SiO_2_-NH_2_/CoSB Modified Carbon Paste Electrode for Simultaneous Detection of Acetaminophen and Chlorpheniramine. Advanced Journal of Chemistry-Section A 4(2) (2021) 152-164. https://doi.org/10.22034/ajca.2021.275901.1246 10.22034/ajca.2021.275901.1246

[37] Mahari S. Gandhi S.. Electrochemical immunosensor for detection of avian Salmonellosis based on electroactive reduced graphene oxide (rGO) modified electrode. Bioelectrochemistry 144 (2022) 108036. https://doi.org/10.1016/j.bioelechem.2021.108036 10.1016/j.bioelechem.2021.108036 34906818

[38] Karimi-Maleh H. Karaman C. Karaman O. Karimi F. Vasseghian Y. Fu L. Baghayeri M. Rouhi J. Senthil Kumar P. Show P.L. Rajendran S.. Nanochemistry approach for the fabrication of Fe and N co-decorated biomass-derived activated carbon frameworks: a promising oxygen reduction reaction electrocatalyst in neutral media. Journal of Nanostructure in Chemistry 12(3) (2022) 429-439. https://doi.org/10.1007/s40097-022-00492-3 10.1007/s40097-022-00492-3

[39] Hosseini Fakhrabad A. Sanavi Khoshnood R. Abedi M.R. Ebrahimi M.. Fabrication a composite carbon paste electrodes (CPEs) modified with multi-wall carbon nanotubes (MWCNTs/N, N-Bis (salicyliden)-1,3-propandiamine) for determination of lanthanum (III). Eurasian Chemical Communications 3(9) (2021) 627-634. DOI: http://dx.doi.org/10.22034/ecc.2021.288271.1182 10.22034/ecc.2021.288271.1182

[40] Yang M. Sun Z. Jin H. Gui R.. Sulfur nanoparticle-encapsulated MOF and boron nanosheet-ferrocene complex modified electrode platform for ratiometric electrochemical sensing of adriamycin and real-time monitoring of drug release. Microchemical Journal 177 (2022) 107319. https://doi.org/10.1016/j.microc.2022.107319 10.1016/j.microc.2022.107319

[41] Farahmandjou M. Khalili P.. ZnO nanoparticles synthesized by co-precipitation method; Morphology and optoelectronic study. Asian Journal of Green Chemistry 5(2) (2021) 219-226. https://doi.org/10.22034/ajgc.2021.261206.1287 10.22034/ajgc.2021.261206.1287

[42] Kumar P.S. Sreeja B.S. Kumar K.K. Padmalaya G.. Static and dynamic analysis of sulfamethoxazole using GO/ZnO modified glassy carbon electrode by differential pulse voltammetry and amperometry techniques. Chemosphere 302 (2022) 134926. https://doi.org/10.1016/j.chemosphere.2022.134926 10.1016/j.chemosphere.2022.134926 35561779

[43] Ismaeel S.A. Al-Bayati Y.K.. Determination of trace metformin in pharmaceutical preparation using molecularly imprinted polymer based pvc-membrane. Eurasian Chemical Communications 3(11) (2021) 812-830. http://dx.doi.org/10.22034/ecc.2021.300477.1224 10.22034/ecc.2021.300477.1224

[44] Dessie Y. Tadesse S.. A Review on Advancements of Nanocomposites as Efficient Anode Modifier Catalyst for Microbial Fuel Cell Performance Improvement. Journal of Chemical Reviews 3(4) (2021) 320-344. http://dx.doi.org/10.22034/jcr.2021.314327.1128 10.22034/jcr.2021.314327.1128

[45] Martins E.C. Santana E.R. Spinelli A.. Nitrogen and sulfur co-doped graphene quantum dot-modified electrode for monitoring of multivitamins in energy drinks. Talanta 252 (2023) 123836. https://doi.org/10.1016/j.talanta.2022.123836 10.1016/j.talanta.2022.123836 35985191

[46] Zare Kazemabadi F. Heydarinasab A. Akbarzadehkhiyavi A. Ardjmand M.. Development, Optimization and in vitro Evaluation of Etoposide loaded Lipid Polymer Hybrid Nanoparticles for controlled Drug Delivery on Lung Cancer. Chemical Methodologies 5(2) (2021) 135-152. https://doi.org/10.22034/chemm.2021.121495 10.22034/chemm.2021.121495

[47] Wang S. Wang H. Liu S. Guo H. Meng J. Chang M. Wu S.. Highly sensitive detection of fluoride based on poly (3-aminophenylboronic acid)-reduced graphene oxide multilayer modified electrode. Food Chemistry 400 (2023) 134042. https://doi.org/10.1016/j.foodchem.2022.134042 10.1016/j.foodchem.2022.134042 36055148

[48] Kavade R. Khanapure R. Gawali U. Patil A. Patil S.. Degradation of Methyl orange under visible light by ZnO-Polyaniline nanocomposites. Journal of Applied Organometallic Chemistry 2(2) (2022) 101-112. http://dx.doi.org/10.22034/jaoc.2022.349558.1056 10.22034/jaoc.2022.349558.1056

[49] Shayegan H. Safarifard V. Taherkhani H. Rezvani M.A.. Efficient removal of cobalt(II) ion from aqueous solution using amide-functionalized metal-organic framework. Journal of Applied Organometallic Chemistry 2(3) (2022) 109-118. DOI: http://dx.doi.org/10.22034/jaoc.2022.154718 10.22034/jaoc.2022.154718

[50] Mazloum-Ardakani M. Beitollahi H. Ganjipour B. Naeimi H.. Novel carbon nanotube paste electrode for simultaneous determination of norepinephrine, uric acid and d-penicillamine. International Journal Electrochemical Science 5 (2010) 531-546.

[51] Duan H. Wang D. Li Y.. Green chemistry for nanoparticle synthesis. Chemical Society Reviews 44(16) (2015) 5778-5792. https://doi.org/10.1039/C4CS00363B 10.1039/C4CS00363B 25615873

[52] Bijad M. Hojjati-Najafabadi A. Asari-Bami H. Habibzadeh S. Amini I. Fazeli F.. An overview of modified sensors with focus on electrochemical sensing of sulfite in food samples. Eurasian Chemical Communications 3(2) (2021) 116-138. DOI: http://dx.doi.org/10.22034/ecc.2021.268819.1122 10.22034/ecc.2021.268819.1122

[53] Abdul Hassan M.M. Hassan S. Hassan K.A.. Green and chemical synthesis of bimetallic nanoparticles (Fe/Ni) supported by zeolite 5A as a heterogeneous Fenton-like catalyst and study of kinetic and thermodynamic reaction for decolorization of reactive red 120 dye from aqueous pollution. Eurasian Chemical Communications 4 (2022) 1062-1086. https://doi.org/10.22034/ecc.2022.342067.1466 10.22034/ecc.2022.342067.1466

[54] Ariavand Sh. Ebrahimi M. Foladi E.. Design and Construction of a Novel and an Efficient Potentiometric Sensor for Determination of Sodium Ion in Urban Water Samples. Chemical Methodologies 6 (2022) 886-904. https://doi.org/10.22034/chemm.2022.348712.1567 10.22034/chemm.2022.348712.1567

[55] Paulchamy B. Arthi G. Lignesh B.D.. A simple approach to stepwise synthesis of graphene oxide nanomaterial. Journal of Nanomedicine & Nanotechnology 6(1) (2015) 1. https://doi.org/10.4172/2157-7439.1000253 10.4172/2157-7439.1000253

[56] Nabi Bidhendi G. Mehrdadi N. Firouzbakhsh M.. Removal of lead from wastewater by iron–benzenetricarboxylate metal-organic frameworks. Chemical Methodologies 5 (2021) 271-284. https://doi.org/10.22034/chemm.2021.130208 10.22034/chemm.2021.130208

[57] Dehno Khalaji A. Mohammadi N. Emami M.. NiO nanoparticles: Synthesis, characterization, and methyl green removal study. Progress in Chemical and Biochemical Research 4(4) (2021) 372-378. https://doi.org/10.22034/pcbr.2021.294420.1194 10.22034/pcbr.2021.294420.1194

[58] Obaid A. Al-ghabban S. Al-Hussain R.. Appraising Antioxidant and Antibacterial Activities of Zinc Oxide Nanoparticles Synthesized Biologically by Iraqi Propolis, Chemical Methodologies 6(5) (2022) 366-371. https://doi.org/10.22034/chemm.2022.332390.1448 10.22034/chemm.2022.332390.1448

[59] Pirozmand M. Nezhadali A. Payehghadr M. Saghatforoush L.. Ultratrace determination of cadmium ion in petro-chemical sample by a new modified carbon paste electrode as voltammetric sensor. Eurasian Chemical Communications 2 (2020) 1021-1032. https://doi.org/10.22034/ecc.2020.241560.1063 10.22034/ecc.2020.241560.1063

[60] Mousavi Ghahfarokhi S.E. Helfi K. Zargar Shoushtari M.. Synthesis of the Single-Phase Bismuth Ferrite (BiFeO3) Nanoparticle and Investigation of Their Structural, Magnetic, Optical and Photocatalytic Properties. Advanced Journal of Chemistry-Section A 5(1) (2022) 45-58. https://doi.org/10.22034/ajca.2021.309069.1284 10.22034/ajca.2021.309069.1284

[61] Qian L. Durairaj S. Prins S. Chen A.. Nanomaterial-based electrochemical sensors and biosensors for the detection of pharmaceutical compounds. Biosensors and Bioelectronics 175 (2021) 112836. https://doi.org/10.1016/j.bios.2020.112836 10.1016/j.bios.2020.112836 33272868

[62] Tallapaneni V. Mude L. Pamu D. Karri V.V.S.R.. Formulation, characterization and in vitro evaluation of dual-drug loaded biomimetic chitosan-collagen hybrid nanocomposite scaffolds. Journal of Medicinal and Chemical Sciences 5 (2022) 1059-1074. https://doi.org/10.26655/JMCHEMSCI.2022.6.19 10.26655/JMCHEMSCI.2022.6.19

[63] Kannan A. Radhakrishnan S.. Fabrication of an electrochemical sensor based on gold nanoparticles functionalized polypyrrole nanotubes for the highly sensitive detection of l-dopa. Materials Today Communications 25 (2020) 101330. https://doi.org/10.1016/j.mtcomm.2020.101330 10.1016/j.mtcomm.2020.101330

[64] Ganesha H. Veeresh S. Nagaraju Y.S. Suresh D.S. Devendrappa H.. Micelles self-degraded polypyrrole nanotube-cobalt oxide nanocomposite based electrochemical sensor for detection of Ascorbic acid. Inorganic Chemistry Communications 145 (2022) 109975. https://doi.org/10.1016/j.inoche.2022.109975 10.1016/j.inoche.2022.109975

[65] Bard A.J. Faulkner L. R., Electrochemical Methods: Fundamentals and Applications, John Wiley & Sons, New York, 2nd edition, 2001.

